# Why Are Viruses Spiked?

**DOI:** 10.1128/mSphere.01339-20

**Published:** 2021-02-17

**Authors:** Chongyang Shen, Scott A. Bradford

**Affiliations:** a Department of Soil and Water Sciences, China Agricultural University, Beijing, China; b USDA, ARS, Sustainable Agricultural Water Systems Unit, Davis, California, USA; University of Michigan-Ann Arbor

**Keywords:** attacking, environment, fusion, receptor-specific interaction, release, spikes, transport, virus

## Abstract

Many viruses, such as severe acute respiratory syndrome coronavirus 2 (SARS-CoV-2) and human immunodeficiency virus (HIV), have a structure consisting of spikes protruding from an underlying spherical surface. Research in biological and colloidal sciences has revealed secrets of why spikes exist on virus surfaces. Specifically, the spikes favor virus attachment on surfaces via receptor-specific interactions (RSIs), mediate the membrane fusion, and determine or change viral tropism. The spikes also facilitate viruses to approach surfaces before attachment and subsequently escape back to the environment if RSIs do not occur (i.e., easy come and easy go). Therefore, virus spikes create the paradox of having a large capacity for binding with cells (high infectivity) and meanwhile great mobility in the environment. Such structure-function relationships have important implications for the fabrication of virus-like particles and analogous colloids (e.g., hedgehog- and raspberry-like particles) for applications such as the development of antiviral vaccines and drug delivery.

## PERSPECTIVE

Many viruses that pose a significant risk to public health have a structure consisting of spikes protruding from an underlying spherical surface, including severe acute respiratory syndrome coronavirus 2 (SARS-CoV-2), influenza virus, and human immunodeficiency virus (HIV) (1, 2). These viruses have a large capacity for binding with cells (high infectivity) and meanwhile great mobility in the environment. Research in biological and colloidal sciences revealed that the spikes on virus surfaces are the culprit that creates this paradox between binding and mobility (3, 4). Specifically, the spikes favor virus attachment to surfaces via receptor-specific interactions (RSIs) and subsequently mediate the membrane fusion. In addition to the RSIs that can occur only at very short separation distances between the virus and surface, the spikes facilitate viruses in approaching surfaces before the attachment and subsequently escaping back to the environment if RSIs do not occur through manipulating long-range colloidal interactions (i.e., easy come and easy go).

Spiked viruses invade their host cells by interaction of their spikes with molecules on surfaces of the cells. For example, spike proteins of coronaviruses can be divided into two important functional subunits, S1 subunits and the S2 domain (which form heads and the stalk of the spikes, respectively) (3, 5). The S1 subunits of coronaviruses recognize and bind to receptors on cellular surfaces, such as angiotensin-converting enzyme 2 (ACE2) for SARS-CoV, dipeptidyl peptidase 4 (DPP4) for Middle East respiratory syndrome coronavirus, and sugar for bovine coronavirus. Subsequently, the S2 subunits undergo conformational changes, causing fusion between the viral envelope and the cell membrane (6). Such RSIs due to the spikes result in viral tropism, and the change of the spike proteins may cause changes in viral tropism. Note that the virus spikes may also be the prime target of the humoral response (7). For example, the coronavirus spike proteins contain major neutralization epitopes, which trigger most of the host immune responses and thus may be used as subunit vaccines against coronavirus infections.

**FIG 1:**
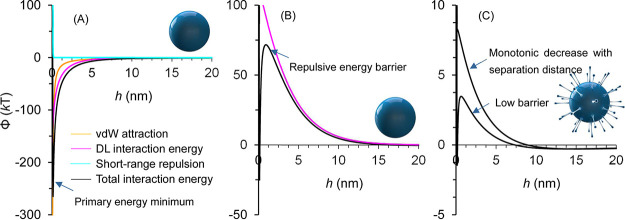
Variations in interaction energy components or total interaction energy (Φ) with separation distance (*h*). (A and B) Interactions of a smooth spherical colloid with a homogeneous surface. The DL interaction is attractive and repulsive due to oppositely and similarly charged colloids and surfaces in panels A and B, respectively. (C) Interaction of a spiked spherical colloid with a homogeneous surface. The primary minimum and energy barrier are low and can even disappear in electrolytes with low ionic strengths, causing a monotonic decease of interaction energy with separation distance.

The spikes not only play a critical role in attachment to host cell surfaces via receptor-specific interactions, as shown above, but they also facilitate the approach of viruses to cell surfaces before the attachment, as revealed by colloid science (4). Specifically, interaction of colloidal particles such as viruses with a surface in electrolyte solution is controlled by colloidal interaction forces. The well-known Derjaguin-Landau-Verwey-Overbeek (DLVO) theory considers the sum of van der Waals (vdW) and double-layer (DL) interactions (8, 9). The vdW attraction increases with deceasing separation distance between a particle and surface (Fig. 1A). The DL interaction is attractive when the particle and surface are oppositely charged in the electrolyte solution. In this case, particles will be forced to approach the surface due to vdW and DL attraction and eventually attach in the primary minimum of the interaction energy curve. The depth of this primary energy minimum is limited due to the existence of short-range repulsion (e.g., hydration, steric, and Born repulsion) at small separation distances.

Both colloidal particles and surfaces (e.g., viruses and cell surfaces) are commonly negatively charged under prevalent biological and environmental conditions (10, 11). In this case, the DL interaction is repulsive, and a repulsive energy barrier exists in the interaction energy curve (Fig. 1B). Virus particles must overcome this energy barrier before they can attach in the primary minimum. An energy barrier of only several *kT*s (where *k* is the Boltzmann constant and *T* is the absolute temperature) can essentially inhibit the attachment of particles to the surface by Brownian diffusion because the rate of attachment decreases exponentially with the magnitude of the energy barrier (12). The energy barrier needs to be comparable to the average kinetic energy of a colloid (1.5 *kT*) in order for it to be overcome by Brownian diffusion. Energy barriers between viruses and surfaces are commonly larger than 10 *kT*s under prevalent biological and environmental conditions (10, 13). Therefore, virus particles can overcome the energy barriers and attach at primary minima only under specific conditions that reduce the DL repulsion, e.g., during charge screening at high electrolyte concentrations or in the presence of surface charge heterogeneity. However, particle attachment has been observed to occur over a broad range of environmental conditions even if they are like-charged (14).

Surface roughness has been identified as a critical factor that facilitates particle attachment in a primary minimum (4, 14, 15). Sharp nanoscale protruding asperities, such as virus spikes, can result in very small energy barriers or even eliminate the energy barrier due to a reduction of DL repulsion under most biological and environmental conditions (Fig. 1C). Hence, spiked viruses readily attach to surfaces via primary-minimum association in solution, except under very extreme conditions (e.g., very low ionic strength or a high solution pH). Note that spikes also decrease the primary energy minimum depth and accordingly the adhesive force that acts on viruses by decreasing the vdW attraction. Weakly attached spiked viruses can easily escape back to solution by only a small disturbance of environmental conditions or even Brownian diffusion. The continuous capture and release (i.e., easy come and easy go) of spiked viruses, such as SARS-CoV-2, thus creates both a high-level attack and mobility. Spiked viruses may be firmly attached only when additional short-range attractions, such as RSIs, exist (resulting in deep primary energy minima).

The primary minimum can even be completely eliminated when repulsion from the bulk particle surface overcomes the attraction between nanoscale asperities and the surface (Fig. 1C) (4). In this case, attachment cannot occur because the particle experiences repulsive forces at all separation distances. The absence of a primary minimum will be more prominent when both interacting surfaces contain nanoscale protruding asperities. This is the reason why spiked viruses and “hedgehog” particles do not aggregate and exhibit high stability in water and many other solvents (16, 17). Coating microparticles with nanoparticles or adding nanoparticles into suspensions of microparticles can enhance the stability of microparticles for a similar reason (18, 19).

The unique attachment/aggregation and detachment/disaggregation behavior of spiked viruses and analogous particles provide insight into the rational fabrication of colloids for specific applications, such as the development of antiviral vaccines, *in situ* soil remediation, and drug delivery. For example, the receptor-binding domains in spike proteins of viruses (e.g., SARS-CoV-2) may serve as targets for developing vaccines to inhibit corresponding infectious diseases (20). In addition, nanomaterials such as nanoscale zerovalent iron (nZVI) have been shown to be very effective for the treatment of contaminants in water. However, the application of nZVI for *in situ* remediation of contaminated soil has been very limited because nZVI readily aggregates and attaches to soil particles. The mobility and efficiency of remediation may be significantly enhanced if nZVI surfaces are functionalized with spikes or nanoscale protruding asperities so that they bind only to selected sites.

In conclusion, our work revealed that the virus spikes play a critical role in virus transmission and infection through manipulating both long- and short-range interactions, which explains the paradox of viruses’ high infectivity and meanwhile great mobility in the environment. Virus spikes facilitate surface contact by reducing or even eliminating repulsive energy barriers under common biological and environmental conditions. Once the contact occurs, the spikes favor virus attachment to surfaces via receptor-specific interactions and subsequently mediate the membrane fusion (resulting in infection). If the RSIs do not occur, the viruses are readily released back into the environment because the spikes reduce the primary-minimum depth or the adhesion that acts on the viruses at surfaces. These findings have important implications for the development of novel technologies for virus disease control (e.g., CoV disease 2019 [COVID-19]).
